# Profile qualitative variables on the dynamics of weight loss programs in dogs

**DOI:** 10.1371/journal.pone.0261946

**Published:** 2022-01-12

**Authors:** Thiago Henrique Annibale Vendramini, Rodrigo Fernando Gomes Olivindo, Rafael Vessecchi Amorim Zafalon, Mariana Fragoso Rentas, Lucca Denuci Zanini, Andressa Rodrigues Amaral, Vivian Pedrinelli, Vinicius Vasques de Oliveira, Larissa Wünsche Risolia, Fabio Alves Teixeira, Márcio Antonio Brunetto

**Affiliations:** 1 Pet Nutrology Research Center, Nutrition and Production Department, School of Veterinary Medicine and Animal Science, University of São Paulo (USP), Pirassununga, Brazil; 2 Veterinary Nutrology Service, Veterinary Teaching Hospital, School of Veterinary Medicine and Animal Science, University of Sao Paulo (USP), Sao Paulo, Brazil; University of Bologna, ITALY

## Abstract

Obesity is the most common nutritional disorder in dogs and it is associated with many comorbidities. Some obesity risk factors have already been established, however, the evaluation of the effect of different individual variables on weight loss induced by calorie restriction, although very important, is still poorly explored. The weight loss protocol can be updated and improved by more precise and adjusted equations throughout the weight loss program in the clinical routine practice. Therefore, the objective of this study was to analyze weight loss program dynamics in groups according to reproductive status, age, body size, and breed, as well as to define more accurately the amount of calories per target metabolic weight throughout the program. Data of 1,053 cases, presented between 2012 and 2019 at the Veterinary Hospital of the School of Veterinary Medicine and Animal Science of the University of São Paulo (FMVZ-USP) were retrospectively analyzed. A total of 77 obese dogs (body condition scores 8/9 or 9/9) of different ages, breeds, sizes, and reproductive status were selected. These dogs did not have any concomitant illnesses and successfully completed the weight loss program. Statistical analysis was performed and values of p≤0.05 were considered significant. The proposed weight loss program was based on an energy restriction protocol where daily energy intake (in kcal) was estimated as 70 *kcal* × *target weight*^0.75^. The target weight (TW) was defined as 80% of the animal’s current weight. The average calorie intake for weight loss (calories x target weight^0.75^) was lower for spayed females (62.36), differing from intact males (66.14) and neutered males (65.41), while intact females (63.66) showed intermediate values without differing between groups (p = 0.015). There were no differences between weight loss calories according to age (p = 0.473) or body size (p = 0.084), allowing the use of the same mathematical equation for intact and neutered dogs; for dogs older than 1 year and of different body sizes. Regarding the breed, the average calorie intake was lower (p = 0.002) in mixed breed dogs (61.54xTW^0.75^) when compared to obesity-prone purebred dogs (64.17xTW^0.75^) and other purebreds (65.27xTW^0.75^). It was concluded that spayed females and mixed breed dogs have greater difficulty in losing weight, that is, they need fewer calories per metabolic body weight for the weight loss program to succeed. A more accurate equation for energy requirement for weight loss can improve chances of success, therefore improving compliance and helping clinical management of obesity in dogs.

## Introduction

Canine obesity is a complex condition with a high prevalence (around 40% to 50%) among the worldwide population of dogs [1–5]. Overweight is associated with certain diseases, such as orthopedic [6–8], cardiovascular [9–13] and respiratory [14–17], metabolic disorders like insulin and leptin resistance [18, 19], hyperlipidemia [20–22], immunoinflammatory response alterations [23], and reduced lifespan [24], which can be partially or completely reversed after weight loss despite reaching ideal body condition score [23]. Additionally, overweight also relates to an increased risk for anesthesia [25].

Previous studies have already identified some factors associated with obesity in dogs such as female gender, neutering, increased age, medium body sized dogs, and specific breeds, being Labrador Retrievers and Beagles the most susceptible [1–3, 22–31]. However, the comparison of the behavior and the dynamics of the weight loss program in these different groups has been insufficiently studied.

The first step to promote weight loss is the establishment of a negative energy balance [32]. The use of mathematical equations that can be customized throughout the program can help achieve success during weight loss plans. The objectives of this study were to evaluate a canine weight loss program to identify the effect of differences in reproductive status, age, body size, and breed and to suggest equations according to these results.

## Material and methods

The experimental procedures were approved by the Animal Use Ethics Committee (AUEC) from the School of Veterinary Medicine and Animal Science of the University of São Paulo (protocol number 4883300921).

Data from 1,053 cases of obesity atendidos from the Nutrition Staff of the Internal Medicine Service of the Veterinary Hospital of the University of Sao Paulo, Brazil, between 2012 and 2019 were retrospectively analyzed. A total of 112 cases of dogs with body condition score ≥ 8/9 [26], with no other diseases and which underwent a weight loss program were selected. Thirty-five dogs (35) were removed from the study because owners did not strictly comply with the proposed dietary management (limited portion size of commercial diet and limitation of treats). Therefore, this study was completed with 77 dogs.

The proposed weight loss program was based on an energy-restriction protocol, in which the daily calorie intake corresponded to 60% of their maintenance energy requirement [33], corresponding to the energy requirement for weight loss (ERWL): 70*kcal* × *target weight*^0.75^ per day [18, 34, 35]. The target weight (TW) was defined as 80% of the animal’s current weight. All cases were managed with different commercial dry extruded diets recommended for the treatment of obesity, as indicated by the manufacturers [32].

In order to avoid insufficient or excessive weight loss and the loss of muscle mass, the minimum and maximum weekly weight loss rates (WWLR) were set between 1 and 2% of current body weight per week [36]. When weight loss was not between the calculated interval, ERWL was reduced or increased in 10% [34, 36].

Animals were reassessed and weighed every two weeks to adjust the weight loss protocol if needed and to ensure that it was strictly followed. Time variables were set at the beginning (0), 30, 60, 90, 120, and 150 days after the start of the weight loss program for comparison purposes.

All weight loss programs were carefully followed by experienced professionals. Therefore, it was possible to plot and determine the weight loss curve and the energy requirement over time using the same equation [17, 18, 34] to determine the amount of calories per TW^0.75^, as well as the development of an adjusted equation based on the results for each one of them.

Dogs were listed in 4 different groups distinguished by reproductive status (neutered male; intact male; spayed female; and intact female), age (from 1 to 8 years old or older than 8 years), breed (mixed breed dogs; purebred dogs popularly known as obesity-prone like Golden Retriever, Labrador, Beagle, English Bulldog, Pug, Cavalier King, Cocker Spaniel, and Dachshund; and purebred dogs not obesity-prone), and body size [small size (up to 15 kg); medium size (15 to 25 kg); large and giant size (over 25 kg)]. The definition of size based on the animal’s weight was adapted from Hosgood & Scholl [37].

Statistical analysis was performed with PROC MIXED from the Statistical Analysis System (SAS) software version 9.3. When differences between the averages were detected, these were compared using the Tukey test. The effects of group (reproductive status, age, body size, and breed), time (the variation throughout the weight loss program), and the interaction between group and time were verified; the interaction between the groups (reproductive status, age, body size, and breed) was not verified due to the variety of combinations and possibilities. For the preparation of the curves and regression analysis, the PROC REG tool of the same statistical software was used. Values of p≤0.05 were considered significant.

## Results

### Reproductive status

Spayed females needed fewer calories per TW^0.75^ when compared to neutered and intact males (p = 0.015). The energy requirement was, on average, 62.36, 65.41, and 66.14 per TW^0.75^ for spayed females, neutered males, and intact males, respectively (Table 1); intact females (63.66 per TW^0.75^) showed intermediate values without differing between groups.

**Table 1:** Calorie intake per target weight^0.75^ according with reproductive status.

Variable	Spayed female	Intact female	Neutered male	Intact male	Average	SEM	*P* ^*1*^		
	(n = 38)	(n = 17)	(n = 14)	(n = 8)			*Reproductive status*	*Time*	Group*time
Total weight loss	62.36^B^	63.66^AB^	65.41^A^	66.14^A^	64.77	0.333	**0.015**	**<0.001**	0.5282
Beginning of weight loss	70	70	70	70	70	0	-	-	-
30 days	66.39	67.16	68.19	67.66	67.03	0.491	-	-	-
60 days	62.61	64.48	65.90	66.00	63.99	0.617	-	-	-
90 days	60.86	62.99	65.27	63.93	62.53	0.799	-	-	-
120 days	58.23	60.59	62.09	65.22	59.96	1.028	-	-	-
150 days	57.37	57.56	60.25	69.94	58.29	1.224	-	-	-

^A-B^Averages in the same line followed by different letters differed by 5% in the Tukey test adjusted by PROC MIXED.

^1^Probability for reproductive status, time, and reproductive status*time interaction.

There was no difference in the interaction between reproductive status and time (p = 0.5282). There was, however, an effect of time (p<0.001) throughout the weight loss program and the need for fewer calories as the program progressed. This result was also verified in other variables: reproductive status, age, body size, and breed.

### Age

In this study, age was not a determining factor for weight loss since no differences were observed for dogs aged 1 to 8 years when compared to dogs older than 8 years (p = 0.473) (Table 2).

**Table 2:** Calorie intake per target weight^0.75^ according with age.

Variable	1 to 8 years (n = 40)	> 8 years (n = 37)	Average	SEM	*P* ^1^		
					*Age*	*Time*	Age*time
Total weight loss	64.60	64.95	64.76	0.333	0.473	**<0.001**	0.587
Beginning of weight loss	70.00	70.00	70.00	0.000	-	-	-
30 days	66.59	67.50	67.03	0.491	-	-	-
60 days	63.10	64.94	63.98	0.617	-	-	-
90 days	62.56	62.50	62.53	0.799	-	-	-
120 days	60.37	59.48	59.96	1.028	-	-	-
150 days	59.17	57.22	58.29	1.225	-	-	-

^1^Probability for effect of age, time, and age*time interaction.

### Body size

There was no effect of size (p = 0.084) and interaction of size and time (p = 0.476) during the evaluation of the weight loss program (Table 3).

**Table 3:** Calorie intake per target weight^0.75^ according with body size.

Variable	Small^1^ (n = 28)	Medium^2^ (n = 12)	Large and giant^3^ (n = 37)	Average	SEM	*P* ^4^		
						*Body size*	*Time*	Body size*time
Total weight loss	64.82	62.51	62.92	64.76	0.333	0.084	**<0.001**	0.476
Beginning of weight loss	70.00	70.00	70.00	70.00	0.000	-	-	-
30 days	67.41	65.34	67.19	67.03	0.491	-	-	-
60 days	65.53	62.60	63.32	63.99	0.617	-	-	-
90 days	64.96	63.22	60.75	62.53	0.799	-	-	-
120 days	61.70	59.37	58.98	59.96	1.028	-	-	-
150 days	59.59	60.23	57.11	58.29	1.225	-	-	-

^1^Small sized animals (up to 15 kg)

^2^Medium sized animals (15 to 25 kg)

^3^Large or giant animals (over 25 kg)

^4^Probability for effect of size, time and interaction size*time.

### Breed

An effect of breed was observed during the weight loss program (p = 0.002), where mixed breed dogs required lower calorie intake (Table 4). The breeds popularly known as prone to obesity [Golden Retriever (n = 6); Labrador Retriever (n = 17); Beagle (n = 2); English Bulldog (n = 2); Pug (n = 2); Cavalier King (n = 1); Cocker Spaniel (n = 1); and Dachshund (n = 2)] showed no differences when compared to the remaining evaluated breeds [Weimaraner (n = 1); Yorkshire (n = 1); Schnauzer (n = 1); Lhasa Apso (n = 2); German Shepherd (n = 1); Shetland Shepherd (n = 1); Pinscher (n = 4); and Poodle (n = 7)]. Also, there was no difference in the breed by time interaction (p = 0.078).

**Table 4:** Calorie intake per target weight^0.75^ according with breed.

Variable	Predisposition to obesity^1^ (n = 33)	Mixed breed (n = 26)	Other purebreds^2^ (n = 18)	Average	SEM	*P* ^3^		
						*Breed*	*Time*	Breed*time
Total weight loss	64.17^A^	61.54^B^	65.27^A^	64.77	0.333	**0.002**	**<0.001**	0.078
Beginning of weight loss	70.00	70.00	70.00	70.00	0.000	-	-	-
30 days	66.85	66.32	68.38	67.03	0.491	-	-	-
60 days	64.64	62.83	64.63	63.99	0.617	-	-	-
90 days	63.08	60.08	64.6	62.53	0.799	-	-	-
120 days	61.61	56.26	62.06	59.96	1.028	-	-	-
150 days	60.68	53.95	62.00	58.29	1.224	-	-	-

^A-B^Averages in the same line followed by different letters differed by 5% in the Tukey test adjusted by PROC MIXED.

^1^Breeds popularly classified as prone to obesity [Golden Retriever (n = 6), Labrador (n = 17), Beagle (n = 2), English Bulldog (n = 2), Pug (n = 2), Cavalier King (n = 1), Cocker Spaniel (n = 1), Dachshund (n = 2)]

^2^Other breeds [Weimaraner (n = 1), Yorkshire (n = 1), Schnauzer (n = 1), Lhasa Apso (n = 2), German Shepherd (n = 1), Shetland Shepherd (n = 1), Pinscher (n = 4), Poodle (n = 7)]

^3^Probability for effect of breed, time and breed*time interaction.

### Adjustment of the weight loss equation based on the weight loss period

**Fig 1:**
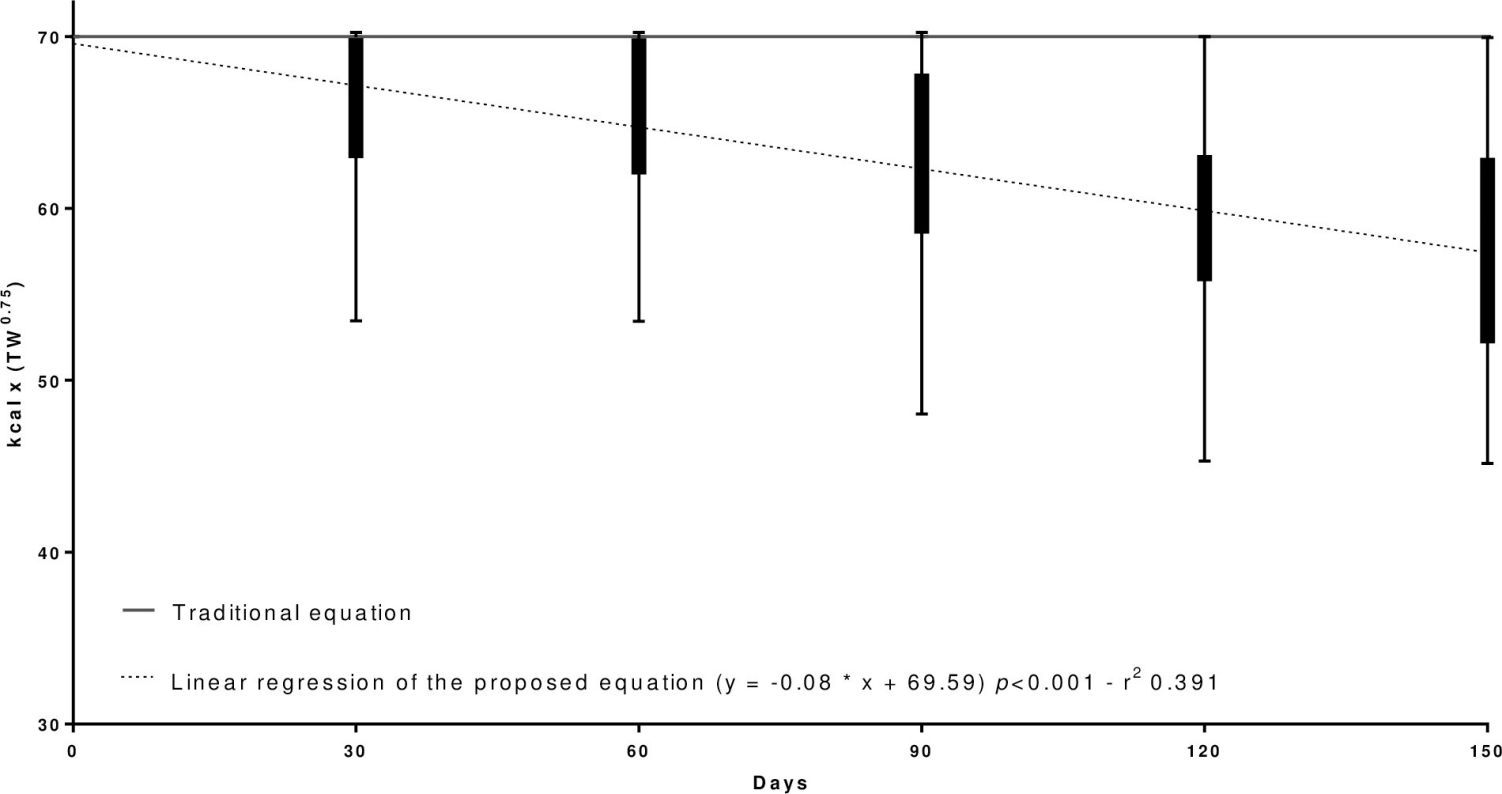
Regression analysis and complete distribution of the general weight loss equation [kcal x (TW^0.75^)] adjusted during the weight loss period.

**Fig 2:**
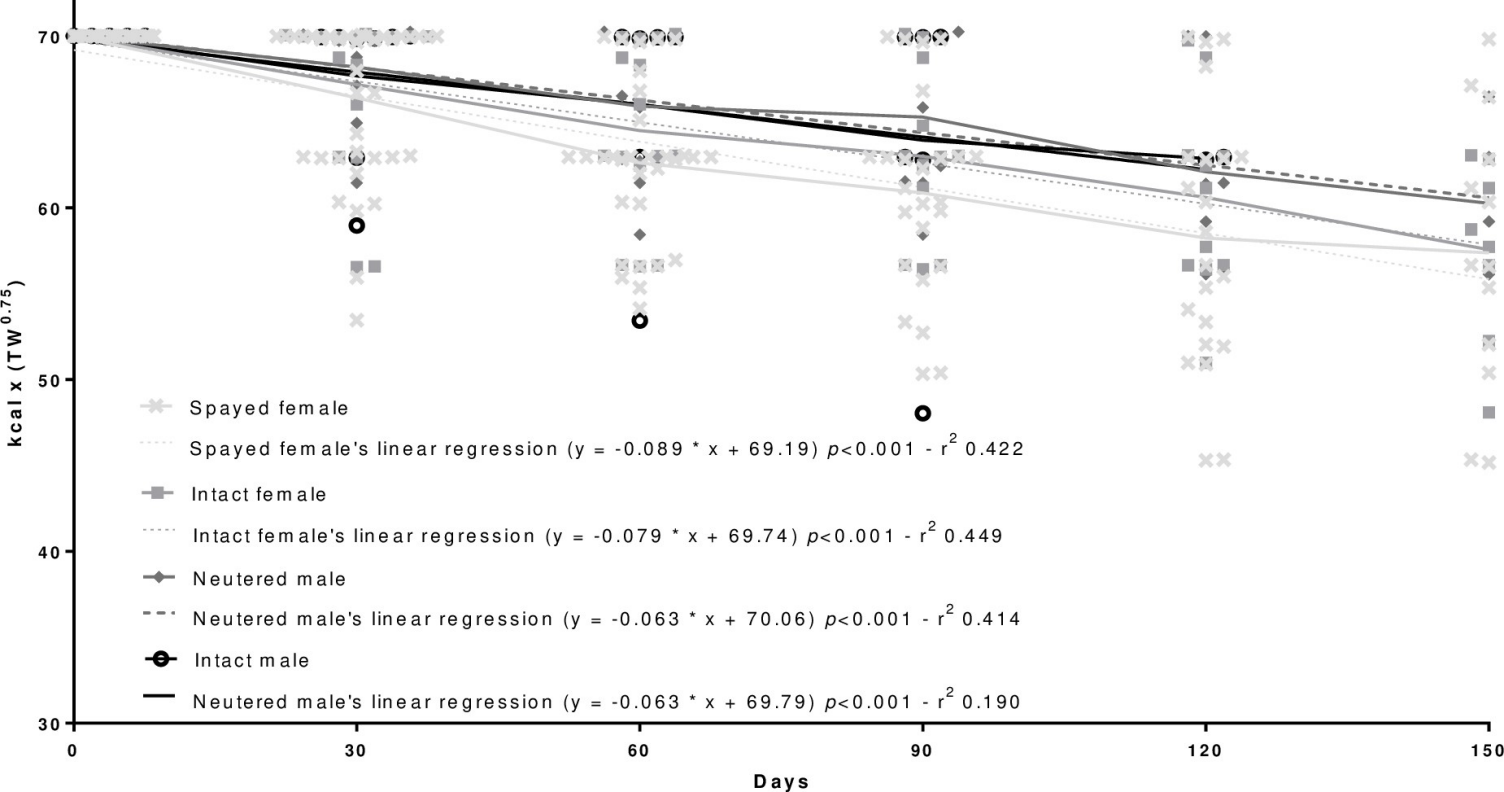
Regression analysis and complete distribution of the calories (kcal) applied to the weight loss equation [kcal x (TW^0.75^)] according with reproductive status.

**Fig 3:**
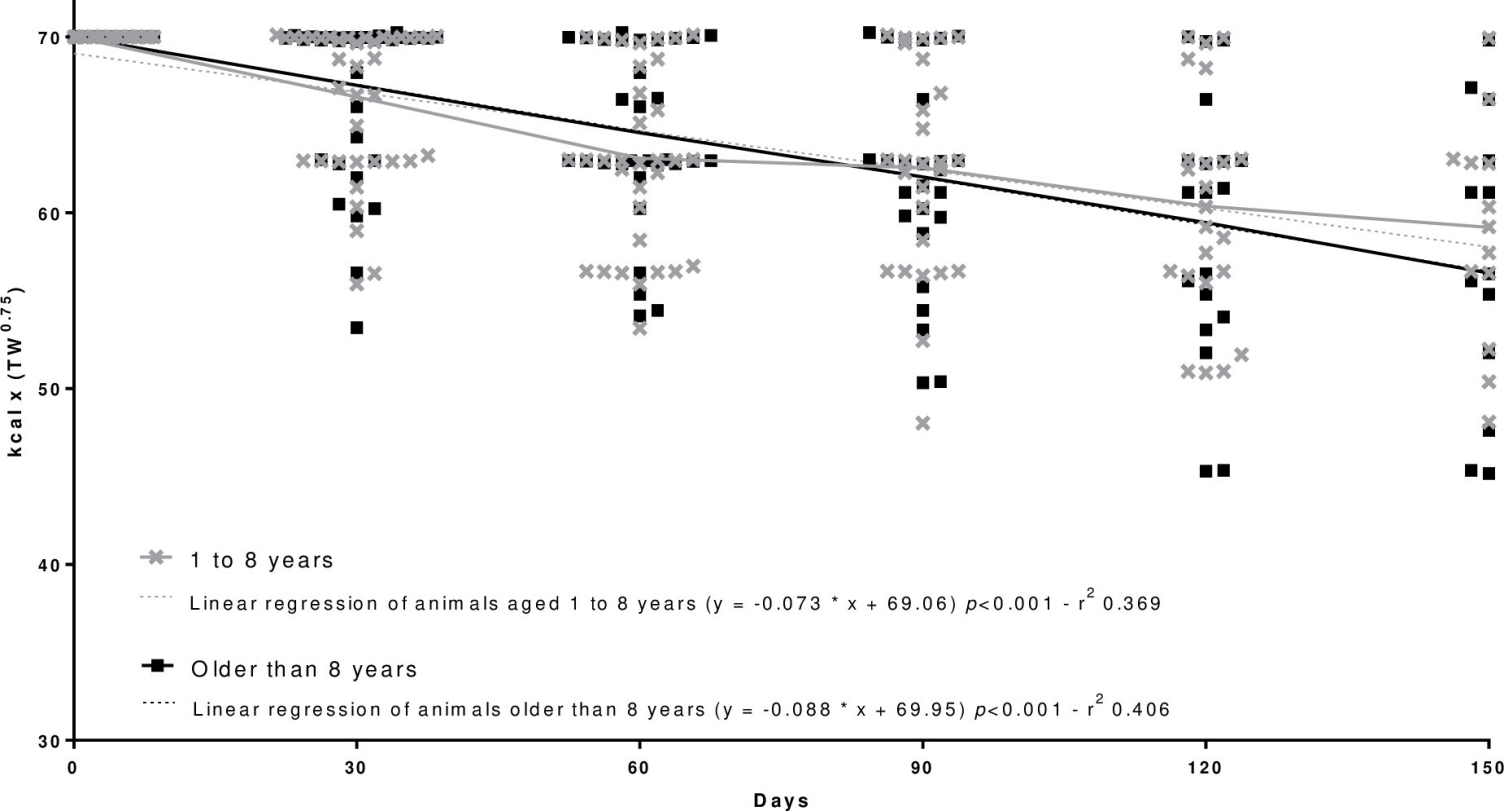
Regression analysis and complete distribution of the calories (kcal) applied to the weight loss equation [kcal x (TW^0.75^)] according with age.

**Fig 4:**
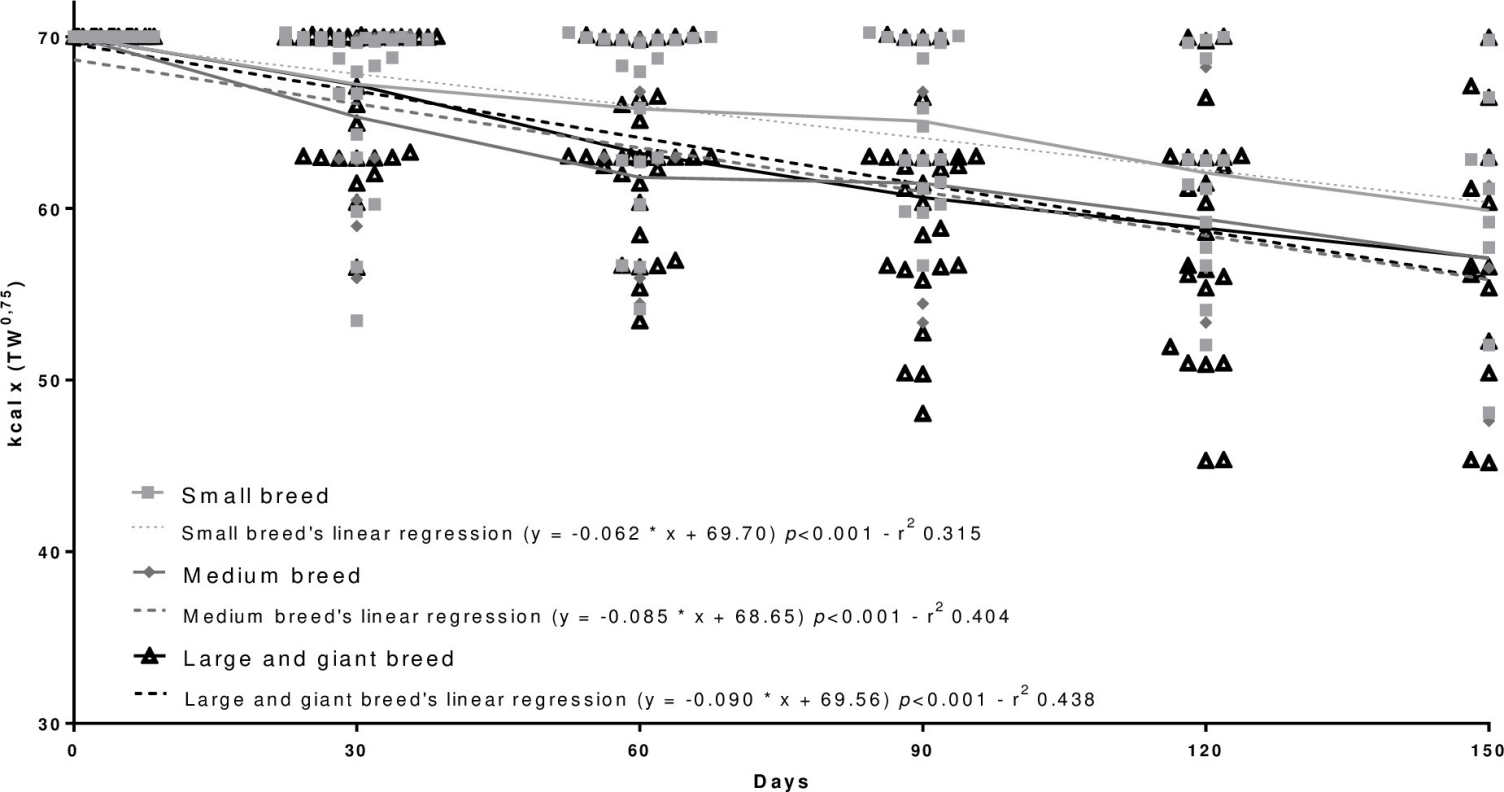
Regression analysis and complete distribution of the calories (kcal) applied to the weight loss equation [kcal x (TW^0.75^)] according with body size.

**Fig 5:**
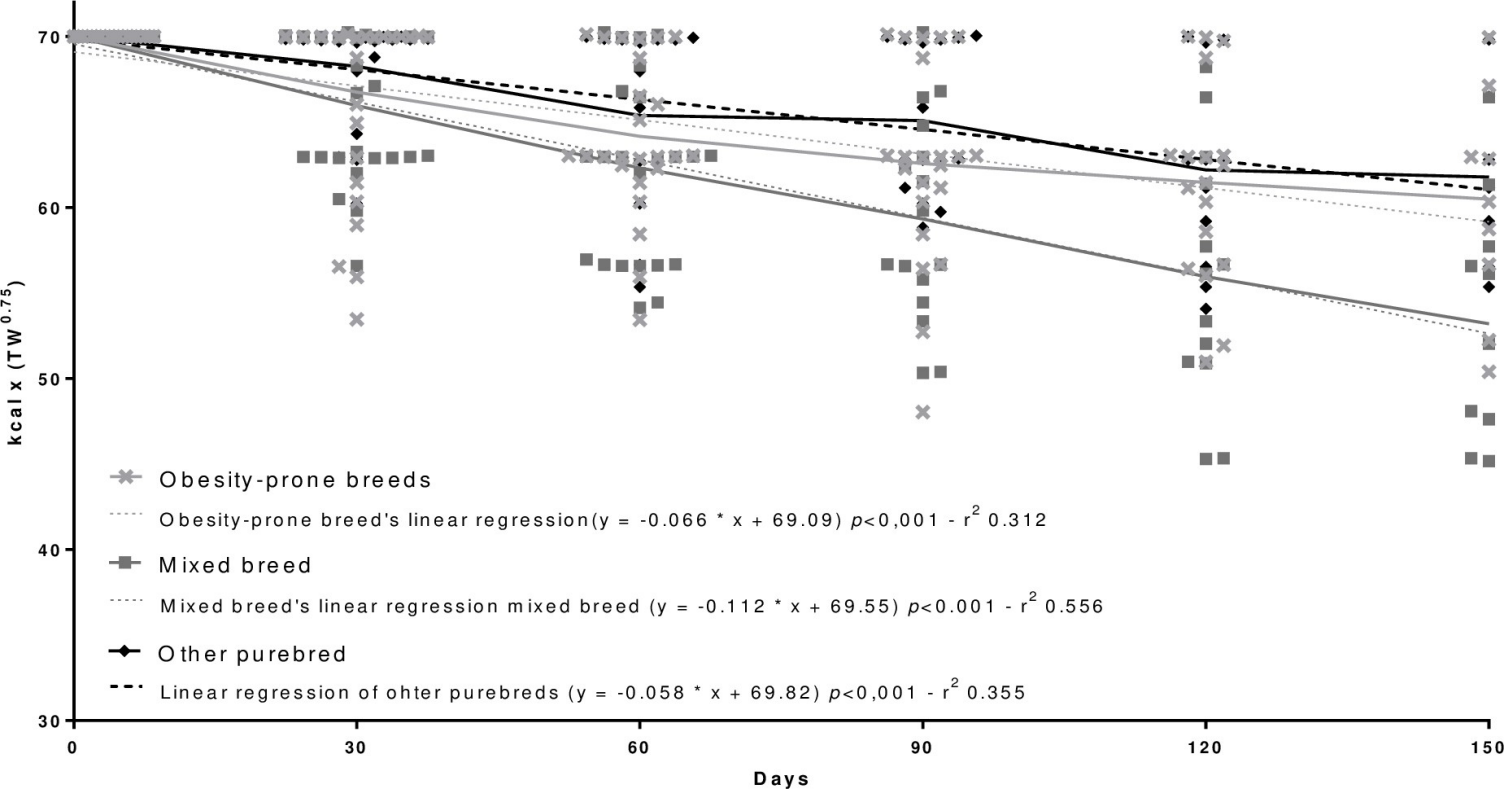
Regression analysis and complete distribution of the calories (kcal) applied to the weight loss equation [kcal x (TW^0.75^)] according with breed.

Regarding the development of a weight loss mathematical equation adjusted for the weight loss period, a general equation for an accurate energy requirement prediction (p<0.001) was proposed (Fig 1). The regression analyses and complete distribution of the kcal x TW^0.75^ according to reproductive status (Fig 2), age (Fig 3), body size (Fig 4), and breed (Fig 5) were also significant (p<0.001).

## Discussion

Females are more susceptible for obesity in the canine population of Australia, United States, France, Sweden, China, Japan, and Spain [1–3, 29–31, 38], and the results are linked to the particularities of the male’s and female’s endocrine system. In this study, it was observed that spayed females required greater calorie restriction to lose weight in a weekly rate of 1 to 2%. This justifies the need to create an adapted equation that fits with the specific requirements to lose weight in an appropriate rate.

In males there is an abundant synthesis and circulation of androgen hormones (testosterone) with anabolic function, which promote an increase in lean mass. The muscle is a tissue with a high metabolic rate that, when associated with the low deposition of adipose tissue, prevents premature aging, locomotor diseases, and delayed decline in the metabolic activity [38]. This suggests that muscle mass helps to maintain an increased energy expenditure and therefore may help to prevent obesity. Furthermore, lean mass stimulates testosterone secretion, resulting in positive feedback. Thus, due to the lack of testosterone in the female, the beneficial effects mentioned above are attenuated, making the weight loss more efficient in male dogs [39].

In human medicine, although several studies also mentioned differences in weight loss success between men and women [40, 41], there is still no consensus on whether and how these physiological differences play a role. The adrenal is an important source of androgens in females [42], which means that even after the procedure for removing the gonads, there is a difference between females and males, which can be enhanced by reducing the energy demand after the procedure.

There are few reports in the literature comparing neutered and intact animals during weight loss programs. Flanagan et al. [43] demonstrated that sexually intact dogs undergoing weight loss programs lost more weight than neutered dogs (p = 0.001), while female dogs lost more weight than male dogs (p = 0.007). McGreevy et al. [29], Colliard et al. [30], Lund et al. [1], Mao et al. [2], Usui et al. [3], and Vendramini et al. [44] observed that neutered animals are more predisposed to obesity. These findings have the same explanation based on the role of the sex hormones because after neutering there is a decrease in circulating anabolic hormones and, consequently, a decrease in the animal’s metabolic rate [39]. Thus, the increase in food intake and the decrease in energy demand after neutering [45] influence the energy mobilization for weight loss in a more impactful way on females. There is also an imbalance between the groups, which can be considered a possible limitation.

Many important physiological changes involving body composition and basal metabolic rate occur with aging [46]. The difference in body composition of dogs can be explained by the increase in the frequency of obesity observed over the years, with body fat increasing from 18% in dogs younger than 1.5 years to 27% in dogs older than seven years [47].

The lower basal metabolic rate may be explained by the decrease in the secretion of anabolic and thermogenic hormones, such as triiodothyronine (T3), thyroxine (T4), growth hormone (GH), and insulin-like growth factor-1 (IGHF1) plus the increased circulation of inflammatory cytokines and physical inactivity [48]. In other studies, age was a risk factor for obesity in dogs [1–3, 29–31]. In this study, age was not a determining factor for weight loss. It is worth mentioning that the dogs included in the study, unlike those included in the above-mentioned studies, were diagnosed only with obesity and not with other comorbidities. The metabolic adaptations of obese animals allow fat to be used as a primary source of energy during the weight loss program, which may differ from obese dogs with chronic diseases. Obesity accompanied by other comorbidities is commonly observed in elderly dogs and decreases the body’s ability to make the necessary metabolic adaptations necessary for a healthy weight loss program, and may use lean mass as source of energy and then compromise the weight loss program [48, 49].

The assessment of the possible relationship between body size and predisposition to obesity is scarce in the literature [3]. Sallander et al. [31] and Usui et al. [3] found, respectively, that large breeds are more predisposed to obesity and that medium-sized breeds are 1.4 times more likely to be obese when compared to miniature-sized dogs. When weight loss is considered, the data available in the literature are very similar to those observed in our study, and there were no differences between the groups (p = 0.084).

The results of the present study partially agree with some results found in the literature regarding breeds. Cocker Spaniels, Cavalier Kings, Dachshunds, and Labrador Retrievers are more prone to obesity [1, 29–31, 50, 51] and, therefore, may have more difficulty losing weight. It is important to note that purebred animals were divided into two groups, one with the breeds most popularly known as predisposed to obesity, and the other with the remaining breeds.

The breed division made in this study was based on popular and unscientific communication and may limit our interpretation of the results. However, as already seen in our results, obesity-prone factors may not necessarily be linked to factors that facilitate or impair weight loss. Therefore, more research is required to answer this question. In addition, the caloric restriction required for weight loss was similar in the purebred groups, but higher in mixed breed dogs, thus demonstrating their difficulty to lose weight when compared to purebred.

The unprecedented assessment of the dynamics of weight loss in different groups compromise the comparisons to the literature, as well as the justification of the findings obtained. One of the possible justifications is based on evolution, which is the gradual genetic change of living organisms over time [52]. Theoretically, animals that spend less energy than others manage to provide a greater reserve for mating and reproduction and have greater reproductive success as a consequence [53]. Thus, in the context of survival and evolution, the greatest difficulty in weight loss is desirable, and it has become an evolutionary step. Mixed breed dogs (any dog who is not purebred and has a combination of two or more lineages of breeds) can still stand out in this context, when compared to breeds genetically selected for other characteristics (such as coat, size, or behavior). Gácsi, McGreevy, Kara & Miklósi [54] also raised an interesting hypothesis involving genetic influences, which states that the current mixed breeds may originate from a population that was under continuous selection for survival skills.

Among all dog breeds included in this study, Labrador Retrievers had the highest documented prevalence of obesity [1, 55], which has been justified recently by Raffan et al. [56], who observed that the deletion of the proopiomelanocortin (POMC) gene is related to the perception of satiety and, consequently, energy intake. Further studies are needed to clarify and contribute to the results, such as those of genomic and metabolomic evaluation of different breeds or mixed breeds. However, they already provided the basis for possible adjustments in the weight loss equations for an animal with greater difficulty to lose weight.

The equation applied in the obesity treatment routine and used by most veterinarians makes an initial proposal for calorie intake [17, 18, 34]. However, adjustments during the weight loss period are almost always necessary. Thus, the evaluation of all cases included in this study also enabled the development of a general ERWL equation based on the period of the weight loss program that the animal currently is, and makes possible the corrections for the previously proposed equation.

The regression analysis performed determined the amount of kcal per TW^0.75^ by the equation y = -0.08 * x + 69.59, where X is the number of days in the weight loss program and Y the amount of calories per kg TW^0.75^. In order to facilitate its use, the equation proposed is kcal/TW^0.75^ = -0.08 * days in weight loss program + 70.

Take for an example a random dog in a weight loss program that returns for a follow-up visit on the 18th day with an inadequate weight loss rate. The equation that best fits the program at that time for an adequate weight loss rate will be: ERWL = (-0.08 * 18 + 70) x target weight^0.75^ = kcal/day. This means that on the 18th day, the veterinarian can adjust the weight loss by predicting the ERWL in a more accurate and precise way, using the personalized equation ERWL = 68.56 x target weight^0.75^. This equation is applicable as a general reference. However, as already described, equations adjusted over the weight loss period by reproductive status, age, body size, and breed were also developed in this study. These can be applied especially in those scenarios where there was a difference between the groups.

Despite the small determination coefficient, justified by the large individual variation of the animals (which is expected in a retrospective study with client-owned dogs), the regression proved to be significant for all dogs and can be a tool for determination of the amount of calories per TW^0.75^ according with the weight loss period of these specific groups that behave more efficiently than the adjustments currently used in current practice. This is a pioneering study in evaluating the behavior and the dynamics of the weight loss program in dogs; and more studies are needed to better understand it.

It must be acknowledged that, as any other study developed with client-owned dogs, the self-reported compliance to the weight loss protocol is an important limitation. Also, as this was a retrospective study, data about exercise was not considered due to inconsistency of this information in dog’s individual files. Furthermore, there was no standardization of the diet of the animals studied, despite all of them having been fed with extruded dry commercial food for weight loss programs. All these factors must be taken into consideration when developing further studies to overcome this problem. However, it must also be taken into consideration the fact that these limitations are a portrait of the real life when there is a team of trained veterinary nutritionists in a veterinary hospital and our results might be more accurate than laboratory and controlled conditions and more accurate for clinical practice.

## Conclusion

In conclusion, females and mixed breed dogs have greater difficulty in losing weight, because they require greater energy restriction for the success of the weight loss program. In addition, the adjusted equation for weight loss, based on the proposed weight loss period, can be a tool to facilitate the clinical routine of the veterinarians, improve compliance of the owner, and benefit the overall health status of the dogs improving performance, time, and weight loss rates.

## References

[1] Lund E, Armstrong P, Kirk C, Klausner J. Prevalence and risk factors for obesity in adult dogs from private us veterinary practices. Int J Appl Res Vet Med. 2006;4: 177.

[2] Mao J, Xia Z, Chen J, Yu J. Prevalence and risk factors for canine obesity surveyed in veterinary practices in Beijing, China. Prev Vet Med. 2013;112: 438–442. doi: 10.1016/j.prevetmed.2013.08.012 24042026

[3] Usui S, Yasuda H, Koketsu Y. Characteristics of obese or overweight dogs visiting private Japanese veterinary clinics. Asian Pac J Trop Biomed. 2016;6: 338–343. doi: 10.1016/j.apjtb.2016.01.011

[4] Montoya-Alonso JA, Bautista-Castaño I, Peña C, Suárez L, Juste MC, Tvarijonaviciute A. Prevalence of canine obesity, obesity-related metabolic dysfunction, and relationship with owner obesity in an obesogenic region of Spain. Front Vet Sci. 2017;4: 59–65. doi: 10.3389/fvets.2017.00059 28487859 PMC5403824

[5] Porsani MYH, Teixeira FA, Oliveira VV, Pedrinelli V, Dias RA, German AJ, et al. Prevalence of canine obesity in the city of São Paulo, Brazil. Sci Rep. 2020;10: 1–15. doi: 10.1038/s41598-019-56847-4 32826948 PMC7442815

[6] Brown DC, Conzemius MG, Shofer FS. Body weight as a predisposing factor for humeral condylar fractures, cranial cruciate rupture and intervertebral disc disease in Cocker Spaniels. Vet Comp Orthop Traumatol. 1996;9: 75–78. doi: 10.1055/s-0038-1632506

[7] Kealy RD, Lawler DF, Ballam JM, Lust G, Smith GK, Biery DN, et al. Five-year longitudinal study on limited food consumption and development of osteoarthritis in coxofemoral joints of dogs. J Am Vet Med Assoc. 1997;210: 222–225. 9018356

[8] Kealy RD, Lawler DF, Ballam JM, Lust G, Biery DN, Smith GK, et al. Evaluation of the effect of limited food consumption on radiographic evidence of osteoarthritis in dogs. J Am Vet Med Assoc. 2000;217: 1678–1680. doi: 10.2460/javma.2000.217.1678 11110459

[9] Edney AT, Smith PM. Study of obesity in dogs visiting veterinary practices in the United Kingdom. Vet Rec. 1986;118: 391–396. doi: 10.1136/vr.118.14.391 3716092

[10] Pereira-Neto GB, Brunetto MA, Sousa MG, Carciofi AC, Camacho AA. Effects of weight loss on the cardiac parameters of obese dogs. Pesqui Vet Bras. 2010;30: 167–171. doi: 10.1590/s0100-736x2010000200012

[11] Pereira-Neto GB, Brunetto MA, Champion T, Ortiz EMG, Carciofi AC, Camacho AA. Avaliação da pressão arterial sistêmica em cães obesos: Comparação entre os métodos oscilométrico e doppler ultrassônico. Pesqui Vet Bras. 2014;34: 87–91. doi: 10.1590/S0100-736X2014001300016

[12] Piantedosi D, Di Loria A, Guccione J, De Rosa A, Fabbri S, Cortese L, et al. Serum biochemistry profile, inflammatory cytokines, adipokines and cardiovascular findings in obese dogs. Vet J. 2016;216: 72–78. doi: 10.1016/j.tvjl.2016.07.002 27687929

[13] Tropf M, Nelson OL, Lee PM, Weng HY. Cardiac and Metabolic Variables in Obese Dogs. J Vet Intern Med. 2017;31: 1000–1007. doi: 10.1111/jvim.14775 28608635 PMC5508341

[14] Hendricks JC. Brachycephalic Airway Syndrome. Vet Clin North Am Small Anim Pract. 1992;22: 1145–1153. doi: 10.1016/s0195-5616(92)50306-0 1523786

[15] German AJ. The growing problem of obesity in dogs and cats. J Nutr. 2006;136: 1940S–1946S. doi: 10.1093/jn/136.7.1940S 16772464

[16] Bach JF, Rozanski EA, Bedenice D, Chan DL, Freeman LM, Lofgren JLS, et al. Association of expiratory airway dysfunction with marked obesity in healthy adult dogs. Am J Vet Res. 2007;68: 670–675. doi: 10.2460/ajvr.68.6.670 17542702

[17] Pereira-Neto GB, Brunetto MA, Oba PM, Champion T, Villaverde C, Vendramini THA, et al. Weight loss improves arterial blood gases and respiratory parameters in obese dogs. J Anim Physiol Anim Nutr (Berl). 2018;102: 1743–1748. doi: 10.1111/jpn.12963 30006938

[18] Brunetto MA, César Sá F, Prudente Nogueira S, de Oliveira Sampaio Gomes M, Gullo Pinarel A, Toloi Jeremias J, et al. The intravenous glucose tolerance and postprandial glucose tests may present different responses in the evaluation of obese dogs. Br J Nutr. 2011;106: S194–S197. doi: 10.1017/S0007114511000870 22005427

[19] Park H-J, Lee S-E, Oh J-H, Seo K-W, Song K-H. Leptin, adiponectin and serotonin levels in lean and obese dogs. BMC Vet Res. 2014;10: 113. doi: 10.1186/1746-6148-10-113 24886049 PMC4030042

[20] Yasuaki S, Katamoto H, Ohashi F. Serum Lipid and Lipoprotein Concentrations in Obese Dogs. J Vet Med Sci. 1995;57: 595–598. doi: 10.1292/jvms.57.595 8519883

[21] Jeusette IC, Detilleux J, Shibata H, Saito M, Honjoh T, Delobel A, et al. Effects of chronic obesity and weight loss on plasma ghrelin and leptin concentrations in dogs. Res Vet Sci. 2005;79: 169–175. doi: 10.1016/j.rvsc.2004.11.012 15924935

[22] Brunetto MA, Nogueira S, Sá FC, Peixoto Ricardo Souza Vasconcellos M, Ferraudo AJ, Carciofi AC. Correspondência entre obesidade e hiperlipidemia em cães. Cienc Rural. 2011;41: 266–271. doi: 10.1590/S0103-84782011005000004

[23] Vendramini THA, Macedo HT, Amaral AR, Rentas MF, Macegoza MV, Zafalon RVA, et al. Gene expression of the immunoinflammatory and immunological status of obese dogs before and after weight loss. PLoS One. 2020;15. doi: 10.1371/journal.pone.0238638 32966299 PMC7510989

[24] Kealy RD, Lawler DF, Ballam JM, Mantz SL, Biery DN, Greeley EH, et al. Effects of diet restriction on life span and age-related changes in dogs. J Am Vet Med Assoc. 2002;220: 1315–1320. doi: 10.2460/javma.2002.220.1315 11991408

[25] Devito FC, Patricio GCF, Flôr PB, Vendramini THA, Amaral AR, Pfrimer K, et al. Comparative study of anaesthesia induction in obese dogs using propofol dosages based on lean body weight or total body weight. Vet Anim Sci. 2020;10. doi: 10.1016/j.vas.2020.100131 32734031 PMC7386691

[26] Development Laflamme D. and validation of a body condition score system for dogs. Canine Pract. 1997;22: 10–15. doi: 10.1016/j.urolonc.2006.12.013 17826654

[27] Robertson ID. The association of exercise, diet and other factors with owner-perceived obesity in privately owned dogs from metropolitan Perth, WA. Prev Vet Med. 2003;58: 75–83. doi: 10.1016/s0167-5877(03)00009-6 12628772

[28] Mawby DI, Bartges JW, d’Avignon A, Laflamme DP, Moyers TD, Cottrell T. Comparison of various methods for estimating body fat in dogs. J Am Anim Hosp Assoc. 2004;40: 109–114. doi: 10.5326/0400109 15007045

[29] McGreevy PD, Thomson PC, Pride C, Fawcett A, Grassi T, Jones B. Prevalence of obesity in dogs examined by Australian veterinary practices and the risk factors involved. Vet Rec. 2005;156: 695–702. doi: 10.1136/vr.156.22.695 15923551

[30] Colliard L, Ancel J, Benet J-J, Paragon B-M, raldine Blanchard G. Risk Factors for Obesity in Dogs in France. WALTHAM Int Nutr Sci Symp. 2006. doi: 10.1093/jn/136.7.1951S 16772466

[31] Courcier EA, Thomson RM, Mellor DJ, Yam PS. An epidemiological study of environmental factors associated with canine obesity. J Small Anim Pract. 2010;51: 362–367. doi: 10.1111/j.1748-5827.2010.00933.x 20402841

[32] NRC. Nutrient Requirements of Dogs and Cats. 1st ed. In: National Research Council, editor. Nutrient Requirements of Dogs and Cats. 1st ed. Washington, D.C.: National Academy Press; 2006. p. 398. doi: 10.17226/10668

[33] NRC. Nutrient Requirements of Dogs and Cats. Nutrient Requirements of Dogs and Cats. 2006. p. 33.

[34] Carciofi AC, Gonçalves KNV, Vasconcellos RS, Bazolli RS, Brunetto MA, Prada F. A weight loss protocol and owners participation in the treatment of canine obesity. Ciência Rural. 2005;35: 1331–1338. doi: 10.1590/s0103-84782005000600016

[35] Brooks D, Churchill J, Fein K, Linder D, Michel KE, Tudor K, et al. 2014 AAHA weight management guidelines for dogs and cats. J Am Anim Hosp Assoc. 2014;50: 1–11. doi: 10.5326/JAAHA-MS-6331 24216501

[36] German AJ, Holden SL, Mather NJ, Morris PJ, Biourge V. Low-maintenance energy requirements of obese dogs after weight loss. Br J Nutr. 2011;106: S93–S96. doi: 10.1017/S0007114511000584 22005443

[37] Hosgood G, Scholl DT. Evalution of age as a risk factor for perianesthetic morbidity and mortality in the dog. J Vet Emerg Crit Care. 1998;8: 222–236. doi: 10.1111/j.1476-4431.1998.tb00128.x

[38] German AJ, Blackwell E, Evans M, Westgarth C. Overweight dogs exercise less frequently and for shorter periods: Results of a large online survey of dog owners from the UK. J Nutr Sci. 2017;6: 1–4. doi: 10.1017/jns.2017.6 28620486 PMC5465938

[39] Zoran DL. Obesity in Dogs and Cats: A Metabolic and Endocrine Disorder. Veterinary Clinics of North America—Small Animal Practice. 2010. pp. 221–239. doi: 10.1016/j.cvsm.2009.10.009 20219485

[40] Cunningham JJ. Body composition as a determinant of energy expenditure: A synthetic review and a proposed general prediction equation. Am J Clin Nutr. 1991;54: 963–969. doi: 10.1093/ajcn/54.6.963 1957828

[41] Couillard C, Mauriège P, Prud’homme D, Nadeau A, Tremblay A, Bouchard C, et al. Plasma leptin concentrations: Gender differences and associations with metabolic risk factors for cardiovascular disease. Diabetologia. 1997;40: 1178–1184. doi: 10.1007/s001250050804 9349599

[42] Santen RJ, Samojlik E, Demers L, Badder E. Adrenal of male dog secretes androgens and estrogens. Am J Physiol 1980;239: 109–112. doi: 10.1152/ajpendo.1980.239.2.E109 7406039

[43] Flanagan J, Bissot T, Hours M-A, Moreno B, Feugier A, German AJ. Success of a weight loss plan for overweight dogs: The results of an international weight loss study. PLoS ONE 2017;12 (9): e0184199. doi: 10.1371/journal.pone.0184199 28886096 PMC5590893

[44] Vendramini THA, Amaral AR, Pedrinelli V, Zafalon RVA, Rodrigues RBA, Brunetto MA. Neutering in dogs and cats: Current scientific evidence and importance of adequate nutritional management. Nutr Res Rev. 2020;33: 134–144. doi: 10.1017/S0954422419000271 31931899

[45] Schauf S, Salas-Mani A, Torre C, Bosch G, Swarts H, Castrillo C. Effect of sterilization and of dietary fat and carbohydrate content on food intake, activity level, and blood satiety–related hormones in female dogs. J Anim Sci. 2016;94: 4239–4250. doi: 10.2527/jas.2015-0109 27898845

[46] Larsen JA, Farcas A. Nutrition of aging dogs. Veterinary Clinics of North America—Small Animal Practice. 2014. pp. 741–759. doi: 10.1016/j.cvsm.2014.03.003 24951344

[47] Hayek MG, Davenport GM. Nutrition and aging in companion animals. J Anti Aging Med. 1998;1: 117–123. doi: 10.1089/rej.1.1998.1.117

[48] Freeman LM. Cachexia and sarcopenia: Emerging syndromes of importance in dogs and cats. J Vet Intern Med. 2012;26: 3–17. doi: 10.1111/j.1939-1676.2011.00838.x 22111652

[49] Laflamme D, Gunn-Moore D. Nutrition of Aging Cats. Vet Clin North Am—Small Anim Pract. 2014;44: 761–774. doi: 10.1016/j.cvsm.2014.03.001 24951345

[50] Corbee RJ. Obesity in show dogs. J Anim Physiol Anim Nutr (Berl). 2013;97: 904–910. doi: 10.1111/j.1439-0396.2012.01336.x 22882163

[51] Sallander M, Hagberg M, Hedhammar T, Rundgren M, Lindberg JE. Energy-intake and activity risk factors for owner-perceived obesity in a defined population of Swedish dogs. Prev Vet Med. 2010;96: 132–141. doi: 10.1016/j.prevetmed.2010.05.004 20542583

[52] Pianka ER. Evolutionary Ecology. 6th ed. Addison-wesley longman. Addison-Wesley Longman; 2000. doi: 10.2307/4645

[53] Garland T, Huey RB, Bennett AF. Phylogeny and Coadaptation of Thermal Physiology in Lizards: A Reanalysis. Evolution (N Y). 1991;45: 1969. doi: 10.1111/j.1558-5646.1991.tb02703.x 28563962

[54] Gácsi M, McGreevy P, Kara E, Miklósi Á. Effects of selection for cooperation and attention in dogs. Behav Brain Funct. 2009;5: 5–31. doi: 10.1186/1744-9081-5-5 19630939 PMC2731781

[55] O’Neill DG, Church DB, McGreevy PD, Thomson PC, Brodbelt DC. Prevalence of disorders recorded in cats attending primary-care veterinary practices in England. Vet J. 2014;202: 286–291. doi: 10.1016/j.tvjl.2014.08.004 25178688

[56] Raffan E, Dennis RJ, O’Donovan CJ, Becker JM, Scott RA, Smith SP, et al. A Deletion in the Canine POMC Gene Is Associated with Weight and Appetite in Obesity-Prone Labrador Retriever Dogs. Cell Metab. 2016;23: 893–900. doi: 10.1016/j.cmet.2016.04.012 27157046 PMC4873617

